# Combination of FOXD1 and Plk2: A novel biomarker for predicting unfavourable prognosis of colorectal cancer

**DOI:** 10.1111/jcmm.17361

**Published:** 2022-05-17

**Authors:** Yaping Zong, Yiming Miao, Wenchang Li, Minhua Zheng, Zhuoqing Xu, Han Gao, Wenqing Feng, Zifeng Xu, Jingkun Zhao, Lifei Shen, Aiguo Lu

**Affiliations:** ^1^ Department of General Surgery Ruijin hospital Shanghai Jiaotong University School of Medicine Shanghai PR China; ^2^ Shanghai Minimally Invasive Surgery Center Ruijin hospital Shanghai Jiaotong University School of Medicine Shanghai PR China; ^3^ Department of Obstetrics and Gynecology Ruijin Hospital Shanghai Jiaotong University School of Medicine Shanghai PR China

**Keywords:** FOXD1, Plk2, biomarker, colorectal cancer, nomogram

## Abstract

Colorectal cancer (CRC) is a worldwide disease with worse survival. Our objective is to identify previously unrecognized prognostic factors to better evaluate disease progression. Seven GEO datasets were collected and analysed using R software, followed by KEGG enrichment analysis and TFs network construction. LASSO‐COX analysis was performed to select the most useful prognostic features. COX model was used to analyse prognostic factors associated with OS. The survival curve was constructed using Kaplan–Meier analysis. A Nomogram model was also constructed to predict prognosis. A total of 3559 differentially expressed genes (DEGs) and 66 differentially expressed transcription factors were identified. FOXD1 was identified as the most differentially expressed factor of TFs covering the most downstream DEGs and independent risk prognostic factor. Next, FOXD1 expression was detected using immunohistochemical staining in 131 CRC patients’ tissue and the association between FOXD1 expression and clinicopathologic features was analysed. High expression of FOXD1 was correlated with TNM stage and pathological differentiation. Multivariate COX regression analyses confirmed that FOXD1 high‐expression, TNM stage and tumour differentiation were independent prognostic risk factor of OS and DFS. Patients with high expression of FOXD1 were more likely to have poor overall survival and disease‐free survival. The combination of FOXD1 and Plk2 which we have previously reported allowed us to predict the survival of post‐surgical CRC patients more accurately, adding to the former prognostic model based on the TNM Stage. The results showed that patients with high expression of both FOXD1 and Plk2 have the worst survival. A combination of FOXD1 and Plk2 can better evaluate patients’ survival.

## 1 INTRODUCTION

Colorectal cancer (CRC) is one of the most widely seen cancer and the third leading cause of cancer‐related death in and across.^1^ While breakthroughs in the diagnosis and therapy of CRC impress us distinctively during the past decades, CRC‐related mortality is still high. CRC is a type of tumour with highly heterogeneous clinical and molecular diversities. Due to the lack of precise biomarkers to predict survival prognosis, about 25% of patients are diagnosed with metastatic CRC.^2^ In addition, the prediction of adjuvant therapy after surgery is also depending on reliable biomarkers. Therefore, the identification of peculiar molecular biomarkers so as to predict patients at high risk of recurrence and metastasis plus to explore molecular‐targeted therapeutic approaches are required to be further understood.

Transcription factors play a crucial role in these biological processes and several transcription factors have been verified as drivers of both invasiveness and drug resistance.^3^ Forkhead box (FOX) proteins, which regulate plenty of cellular pathways during cancer evolution such as TGF‐βpathway, Wnt pathway, and mitogen‐activated protein kinase pathway, consist of a superfamily of evolutionary‐conserved transcriptional factors.^4^ Accumulating evidence indicates these FOX proteins may act as key “nodes” in cellular networks, enabling cross‐talk among biological pathways. FOXD1 is a member of the Forkhead family.^5^ Some studies have revealed its function in promoting cancer cell proliferation in nasopharyngeal cancer and non‐small cell lung carcinoma.^6^, ^7^ Also, FOXD1 can promote breast cancer progression and drug resistance by inhibiting p27.^8^ However, the role of FOXD1 in CRC remains obscure especially as a biomarker. Our study aims to figure out the prognostic value of FOXD1 in patients with CRC.

Here in recent studies, the expression of FOXD1 was measured in CRC specimens. Next, the correlation between FOXD1 and CRC patients’ clinical and pathological characteristics was also investigated. Finally, we constructed a nomogram prediction model and demonstrated the combination of FOXD1 and Plk2, a protein we have described before,^9^ has more potent power to predict CRC patients’ prognosis.

## 2 MATERIALS AND METHODS

### 2.1 Patients and follow‐up

Altogether 131 post‐surgical CRC patients who underwent operations between 2009 and 2012 in Shanghai Ruijin Hospital (Shanghai, China) were enrolled in this retrospective study. Tumour specimens and paired normal adjacent specimens were collected during operation. A CRC tissue microarray (TMA) was set up using the tissues collected above. The construction of TMA has been approved by the Ethics Committee of Ruijin Hospital (Shanghai, China) and all patients were fully informed. We also gathered Clinical and pathological information such as TNM stage, CEA level, histology, gender, age, tumour size, and tumour location. The follow‐up data were acquired through telephone calls, outpatient visits, or office visits.

### 2.2 Immunohistochemical analysis

Fixation of collected fresh samples was immediately performed by 4% formaldehyde. Then these tissues were embedded in paraffin and dissected to manufacture TMA. TMA staining was performed as described before.^10^ Briefly speaking, the microarray was dewaxed and hydrated first. Citrate buffer (10 mM citric acid, pH 6.0) was used to perform antigen retrieval in the microwave. A 5% animal serum (5%) was used to block the microarray and then the microarray was incubated by primary antibodies including FOXD1 (1:40, Proteintech) and Plk2 (1:200, Santa Cruz) overnight at 4℃. HRP labelled secondary antibody was used to further stain the microarray for 10 min at 37℃. DAB was used to visualize the specimens and hematoxylin was used to counterstain nuclei.^10^

### 2.3 Immunohistochemical score

Two independent pathologists scored the density of immunohistochemical staining of FOXD1 and Plk2 in tumour tissues according to the IRS system.^10^ Scoring for percentage of immunoreactive cells was: 0% (0), 1%–10% (1), 11%–50% (2), 51%–80% (3) and over 80% (4). Scoring for staining intensity: 0 stands for no staining, 1 stands for weak staining, 2 means moderate staining and 3 means intense staining. A single score ranging from 0 to 12 was yielded by multiplying these two types of values for each case. For statistical analysis, cases were categorized as either negative (score 0–3) or positive (score ≥3). For survival analysis on the basis of the combination of FOXD1 and Plk2, tumour tissues with FOXD1 and Plk2 being both positively stained were treated as positive staining (FOXD1^high^/Plk2^high^) group, those with FOXD1 positive staining/Plk2 negative staining or FOXD1 negative staining/Plk2 positive staining were treated as intermediate staining (FOXD1^high^/Plk2^low^ or FOXD1^low^/Plk2^high^) group, and those with both FOXD1 and Plk2 negative staining were treated as negative staining (FOXD1^low^/Plk2^low^).

### 2.4 Kaplan–Meier and nomogram curve construction

Patients’ survival rate was calculated and the survival curve was plotted using Kaplan–Meier analysis in GraphPad Prism 7.0. A predictive nomogram model applying certain factors selected by the multivariate Cox regression analysis was built using R software.

### 2.5 Data source

The gene expression profiles of GSE23878, GSE4107, GSE41328, GSE33113, GSE18088, GSE30540 and GSE31595 were downloaded from the GEO database (http://www.ncbi.nlm.nih.gov/geo). All of these datasets established on the GPL570‐55999 platform (Affymetrix Human Gene Expression Array) contain 50 samples from normal tissues and 272 samples from tumour tissues. In addition, all of these datasets are the expression profile of whole‐genome sequencing and have never been pretreated by any drugs.

### 2.6 Identification of DEGs

The recognition process of the differentially expressed genes (DEGs) was performed using the Linear Models for Microarray Data (Limma) package in R. Criteria of DEGs screen was set as adjusted *P* value <0.05 and log fold‐change (|log2FC|) values ≥2. The DEGs expression between tumour samples and adjacent normal samples were processed to plot heatmap and volcano picture using R. In addition, KEGG pathway enrichment analysis and hierarchical clustering were accomplished using the online tools Sangerbox (http://sangerbox.com/Signin).

### 2.7 CRC‐specific transcriptional regulatory network construction

The human transcription factors list and Position Weight Matrix data were downloaded from JASPAR (http://jaspar.genereg.net/). The promoter sequence file was generated using Python from gencode.v22.annotation. Transcription factors binding sites were calculated by FIMO software in python through a combination of human transcription factors list, Position Weight Matrix data and promoter sequence. Then, we collected differentially expressed transcription factors and genes regulated by these transcription factors from DEGs using R. After removing unnecessary info, the CRC‐specific transcriptional regulatory network was built using the Cytoscape software (http://www.cytoscape.org/).

### 2.8 Selection of transcription factors using the LASSO Cox regression model

LASSO Cox regression model was used to select the most useful prognostic features out of the ten transcription factors. The “glmnet’’ package was used to perform the LASSO Cox regression model analysis.

### 2.9 Statistical analysis

The data were assessed using SPSS 25.0 (SPSS Inc., Armonk, NY, United States) and R3.6.3 (http://www.r‐project.org/). Continuous variables in different subgroups were analysed using an unpaired t‐test and one‐way analysis of variance. The Chi‐square test or Fisher exact test was used to assess the correlation between FOXD1 expression and the clinicopathological characteristics. Kaplan–Meier survival curves with 95% confidence intervals (CIs) are used to map OS and RFS, and the log‐rank test is applied to distinguish differences between subgroups. Independent prognostic factors were sorted through univariate and multivariate cox regression, with *p* < 0.05 as the criterion for variable extraction when performing backward stepwise selection. The concordance index (C‐index) and Akaike information criterion (AIC) were used to measure the accuracy of predictive models. All tests were two‐sided with *α* = 0.05.

## 3 RESULTS

### 3.1 DEGs in CRC and FOXD1 act as one of the top 10 transcription factors covering the most downstream DEGs

**FIGURE 1:**
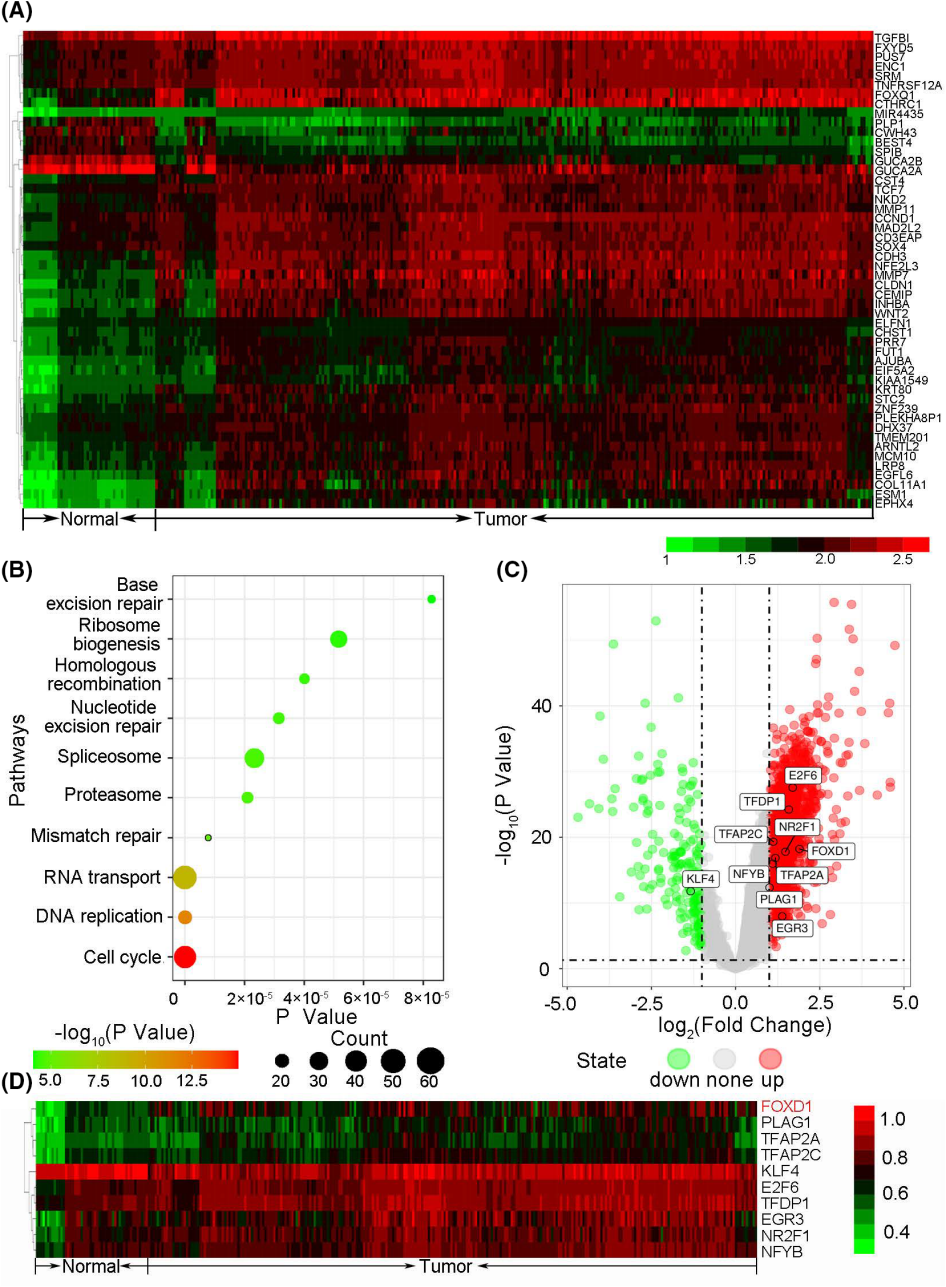
FOXD1 is the most differentially expressed transcription factor in the top 10 TFs covering the most downstream DEGs. A. Heatmap of differentially expressed genes between tumour and normal tissues. B. The top 10 most significantly enriched KEGG pathways of DEGs in CRC. Size of the circle represents a number of genes enriched in this pathway. C. The volcano plot of DEGs showing FOXD1 and other 9 TFs covering the most downstream DEGs. D. Heatmap of the top 10 TFs covering the most downstream DEGs. FOXD1 is the most differentially expressed transcription factor of the 10 TFs

**FIGURE 2:**
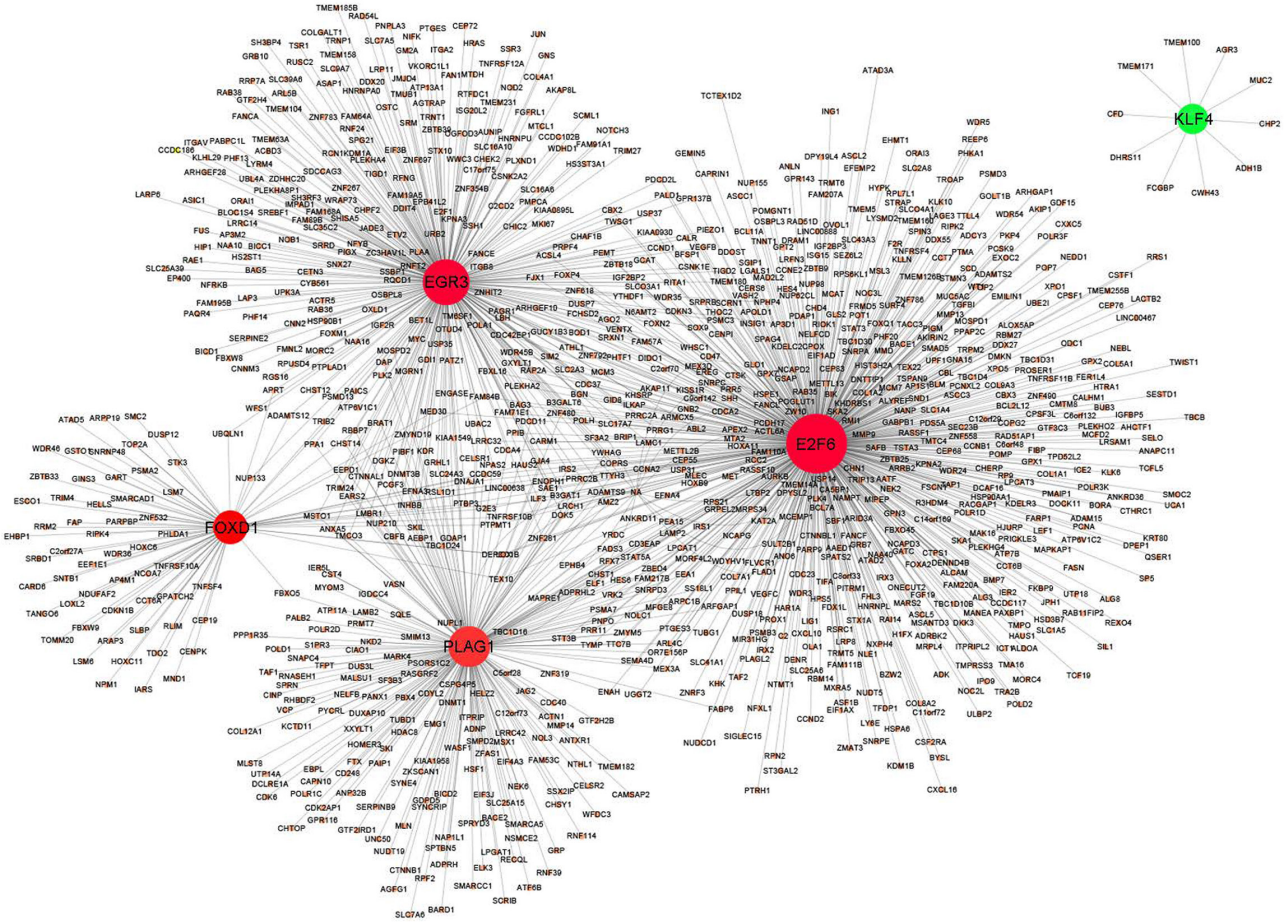
The CRC‐specific transcriptional regulatory network. Green nodes represent downregulated TF; the red nodes represent upregulated TFs. The lines indicated TF‐DEG pairs

Seven datasets (GSE23878, GSE4107, GSE41328, GSE33113, GSE18088, GSE30540 and GSE31595) were enrolled in our study, and basic clinical characteristics were shown in Supporting Information Table S1. The tumour TNM stage and ethnicity of enrolled CRC patients are stages II and III and Caucasian. Compared with normal tissues, altogether 3559 DEGs were sorted out, including 3372 upregulated genes (LogFC ≥ 1) and 187 downregulated genes (LogFC ≤ −1). The top 50 upregulated and downregulated DEGs (sorted by FDR) are presented in the heatmap and volcano plot in Figure 1A,B. Certain DEGs were enriched generally in cell cycle (FDR = 1.13E‐15), DNA replication (FDR = 2.67E‐12) and RNA transport (FDR = 1.15E‐09) pathways according to KEGG enrichment analysis. The top 10 most significantly enriched KEGG pathways for DEGs are shown in Figure 1B and Supporting Information Table S2. In addition, 66 differentially expressed transcription factors which include 62 upregulated and 4 downregulated TFs were identified. Among them, E2F6, PLAG1, EGR3, FOXD1, KLF4, NR2F1, TFAP2A, NFYB, TFAP2C and TFDP1 were the top 10 TFs involved with most downstream DEGs. FOXD1 was the most DEGs in the 10 TFs (Figure 1C,D). Finally, a CRC‐specific transcriptional regulatory network of the top 5 TFs was constructed (Figure 2).

### 3.2 LASSO‐COX regression analysis

**FIGURE 3:**
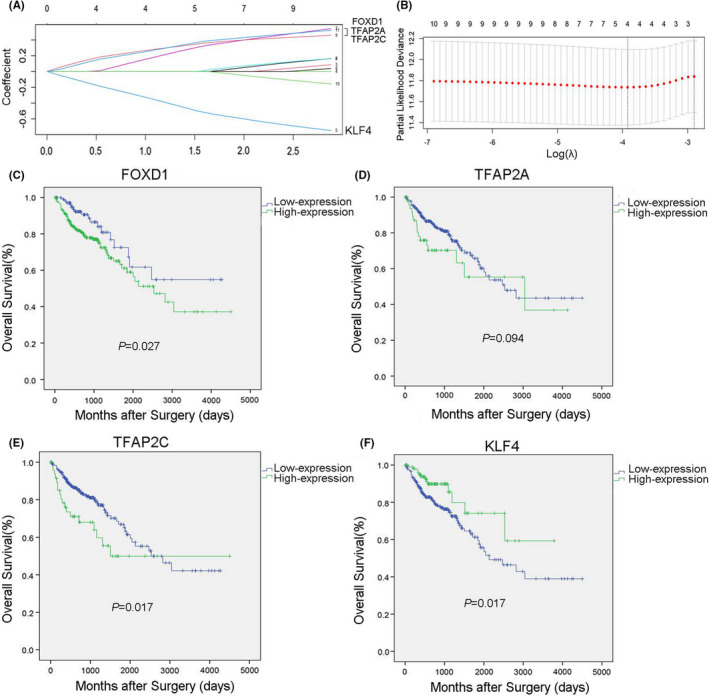
A: LASSO coefficient profiles of the 10 transcription factors. B: Kaplan–Meier survival curve for OS of 4 TFs: FOXD1, TFAP2A, TFAP2C and KLF4 in CRC patients using data from TCGA

The logistic LASSO model is a shrinkage method that can actively select from a large and potentially multicollinear set of variables in the regression, resulting in a more relevant and interpretable set of predictors. We used the LASSO‐Cox regression model to select the most useful prognostic features out of the ten transcription factors and found that FOXD1 (Coefficient, 0.34), TFAP2A (Coefficient, 0.27), TFAP2C (Coefficient, 0.32) and KLF4 (Coefficient, −0.45) (Figure 3A,B). In addition, we established Kaplan–Meier survival curve for OS and found that high expression of FOXD1, as well as TFAP2C, predicts poor OS in CRC patients. Inversely, the high expression of KLF4 predicts better OS in CRC patients (Figure 3C–F). The multivariate COX regression analysis of these four TFs indicated that FOXD1 (*p* = 0.036) was an independent risk prognostic factor and KLF4 (*p* = 0.010) was an independent protective prognostic factor associated with OS (Table 1). Therefore, we choose FOXD1 as a target TF for further analysis and verification.

**TABLE 1:** Multivariate Cox regression analyses of OS with four TFs selected by LASSO analysis using data from TCGA^*^

Transcription factors (*n* = 410)	OS		
	HR	95%CI	*p* value
FOXD1 (high/low)	1.768	1.039–3.009	**0.036**
TFAP2A (high/low)	1.684	0.961–2.950	0.068
TFAP2C (high/low)	1.579	0.935–2.668	0.088
KLF4 (high/low)	0.450	0.245–0.826	**0.010**

* We just get OS data from the TCGA database.

### 3.3 Expression of FOXD1 and its clinicopathologic significance within post‐surgical CRC patients

**FIGURE 4:**
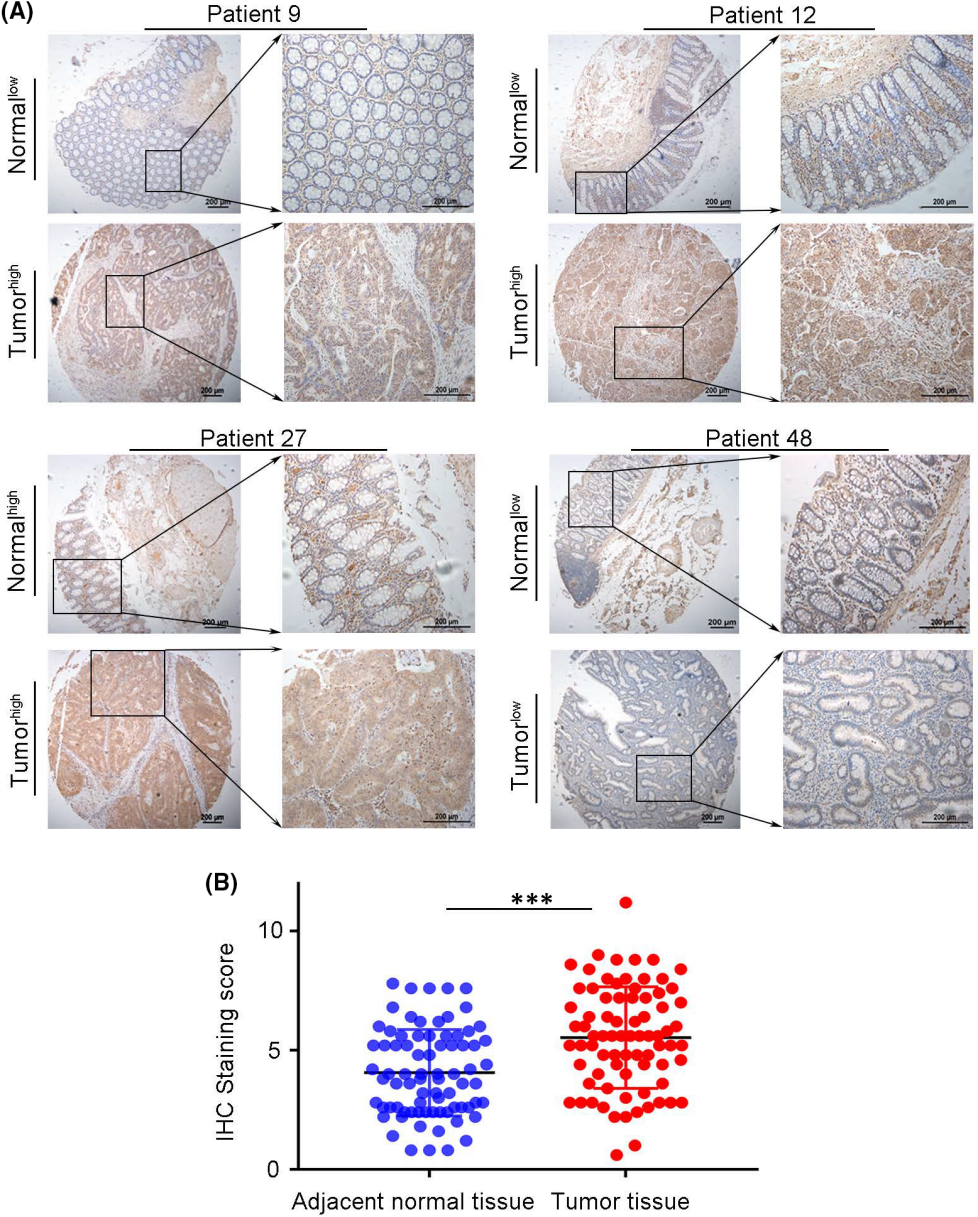
Expression of FOXD1 in CRC tissue. A: Immunohistochemical results showing high expression of FOXD1 in CRC tissues. B: The difference between tumour and peritumoral normal tissues is statistically significant (***p < 0.001)

To further verify the role of FOXD1 in CRC patients’ prognosis, immunochemistry is applied to analyse the TMA, containing 131 pairs of cancer and matched noncancer tissue. The FOXD1 expressions in tumour tissues exceeded those in adjacent normal tissues significantly (Figure 4A,B). Overexpression of FOXD1 was observed in the cytoplasm of tumour cells (Figure 3A). In these cases, the positive expression of FOXD1 was observed in 96 (73.3%) of the tumour specimens, while only 35 (26.7%) of the adjacent normal specimens presented a positive signal (*p* < 0.001) (Figure 4B). In addition, GEO dataset GSE9452 was used to study the expression of FOXD1 in the IBD condition. Bioinformatic analysis showed that expression of FOXD1 has no significant difference in IBD compared with normal tissue. Results were shown in Supplementary Figure 2. Next, the correlation between FOXD1 expression and clinical features in CRC patients were analysed. Results illustrated that FOXD1 high‐expression tumour samples showed a significant correlation with pathological differentiation (*p* = 0.021). However, no significant association was detected between high expression of FOXD1 and age, gender, tumour location or tumour size (Table 2). Meanwhile, the expression of PLK2 in colon and rectum cancer had no significant difference (Table S3).

**TABLE 2:** The correlation between FOXD1 expression and clinicopathologic variables in CRC patients

Clinicopathologic parameters		FOXD1 expression			*p*‐value
		Case (*n* = 131)	Positive	Negative	
Tissues	Carcinoma	131	96	35	<0.001
	Normal tissues	131	66	65	
Age	≥65	63	46	17	0.947
	<65	68	50	18	
Gender	Male	69	47	22	0.159
	Female	62	49	13	
Tumour location	Rectum	48	32	17	0.111
	Colon	83	64	18	
Tumour size	≥5 cm	57	45	12	0.198
	<5 cm	74	51	23	
Differenciation	Well to moderate	88	59	29	0.021
	Poor	43	37	6	
TNM stage	I, II	64	49	15	0.407
	III, IV	67	47	20	

### 3.4 Overexpression of FOXD1 is an independent prognostic factor for post‐surgical patients with CRC

Next we figured out whether FOXD1 expression was an independent risk factor for CRC patients’ prognosis. Univariate Cox regression analysis identified TNM stage (OS: *p* < 0.001; DFS: *p* < 0.001), tumour differentiation (OS: *p* < 0.001; DFS: *p* < 0.001) and positive FOXD1 expression (OS: *p* = 0.033; DFS: *p* = 0.027) as clinicopathological factors that might stronly affect prognosis (Table 3). Variables relevant to CRC outcomes on univariate analysis were included in a multivariate analysis (Table 4). The multivariate analysis indicated that TNM stage (*p* = 0.001), tumour differentiation (*p* = 0.027) and positive FOXD1 expression (*p* = 0.044) were independent prognostic factors associated with OS. Meanwhile TNM stage (*p* < 0.001), tumour differentiation (*p* = 0.004) and FOXD1 expression (*p* = 0.041) were independent prognostic factors for 5‐year DFS in post‐surgical CRC patients.

**TABLE 3:** Univariate Cox regression analyses of OS and RFS with clinicopathologic characteristics in post‐surgical CRC patients

Factor (*n* = 131)	OS			DFS		
	HR	95%CI	*p* value	HR	95%CI	*p* value
Age (<65, ≥65)	1.112	0.562–2.202	0.760	0.901	0.495–1.642	0.735
Gender (female/male)	0.927	0.468–1.834	0.827	0.689	0.377–1.258	0.225
Tumour location (colon/rectum)	0.495	0.223–1.099	0.084	0.763	0.403–1.444	0.406
Tumour size (<5 cm/≥5 cm)	1.387	0.700–2.746	0.348	1.663	0.913–3.029	0.096
Differenciation (well to moderate/poor)	3.939	1.956–7.932	<0.001	4.186	2.267–7.729	**<0.001**
TNM stage (I,II/III,IV)	5.402	2.227–13.101	<0.001	5.483	2.540–11.838	**<0.001**
FOXD1 expression(negative/positive)	3.111	1.093–8.853	0.033	2.645	1.116–6.268	**0.027**

Note

Bold values are statistically significant (*p* < 0.05).

Abbreviations: CI, confidence interval; DFS, disease‐free survival; HR, hazard ratio; OS, overall survival; TNM, tumour node metastasis (8th edition).

**TABLE 4:** Multivariate Cox regression analyses of OS and DFS with clinicopathologic characteristics in post‐surgical CRC patients

Factor (*n* = 131)	OS			DFS		
	HR	95%CI	*p* value	HR	95%CI	*p* value
TNM stage (I,II/III,IV)	4.904	1.961–12.263	0.001	4.889	2.213–10.800	**<0.001**
Differenciation (well to moderate/poor)	2.315	1.103–4.863	0.027	2.587	1.352–4.948	**0.004**
FOXD1 expression(negative/positive)	3.045	1.031–8.990	0.044	2.539	1.037–6.216	**0.041**

Note

Bold values are statistically significant (*p*<0.05).

Abbreviations: CI, confidence interval; HR, hazard ratio; OS, overall survival; RFS, recurrence‐free survival; TNM, tumour node metastasis.

### 3.5 Overexpression of FOXD1 was relevant to the poor OS and DFS

**FIGURE 5:**
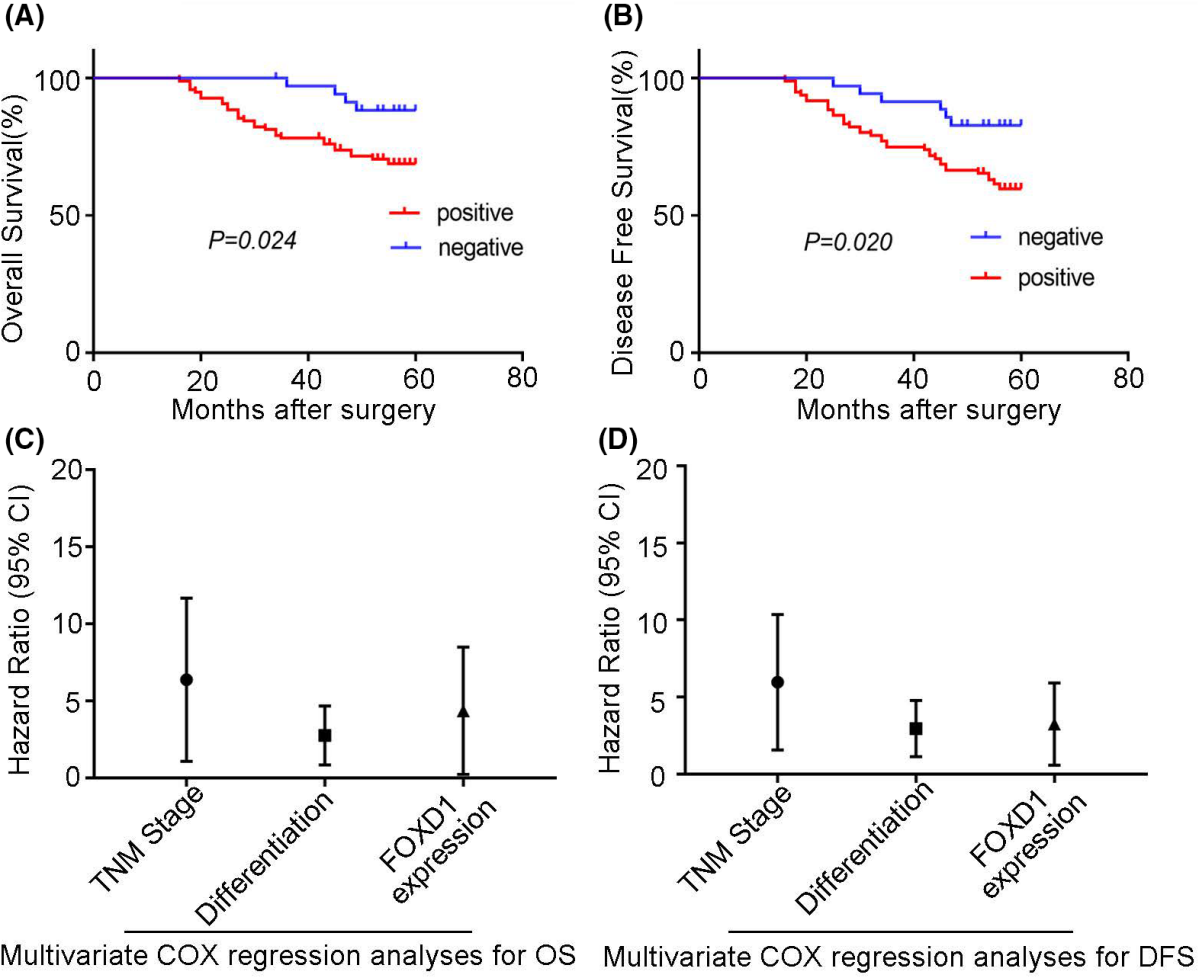
(A) Comparison of overall survival in FOXD1 positive and FOXD1 negative groups. Kaplan–Meier analysis of overall survival in post‐surgical patients with CRC. Blue: patients with negative FOXD1 expression and Red: patients with positive FOXD1 expression. (B) Comparison of recurrence‐free survival in FOXD1 positive and FOXD1 negative groups. Kaplan–Meier analysis of recurrence‐free survival in post‐surgical patients with CRC. Blue: patients with negative FOXD1 expression and Red: patients with positive FOXD1 expression. (C) Forest plot of multivariate Cox regression analysis for OS. (D) Forest plot of multivariate Cox regression analysis for DFS

Kaplan–Meier analysis revealed that 5‐year overall survival rates of FOXD1‐Negative patients were significantly higher than those of the FOXD1‐Positive group (88.2% vs 68.8%). Patients in the FOXD1 positive expression group (*n* = 96) had a significantly poorer overall survival than those in the FOXD1 negative expression group (*n* = 35; *p* = 0.024; Figure 5A). Additionally, patients with negative FOXD1 expression had better recurrence‐free survival than those with FOXD1 positive expression (82.9% vs 59.7%). FOXD1‐Positive expression might indicate less disease‐free survival time (*p* = 0.020, Figure 5B).

### 3.6 The power of the combination of FOXD1 and Plk2 to predict CRC patients’ prognosis

**FIGURE 6:**
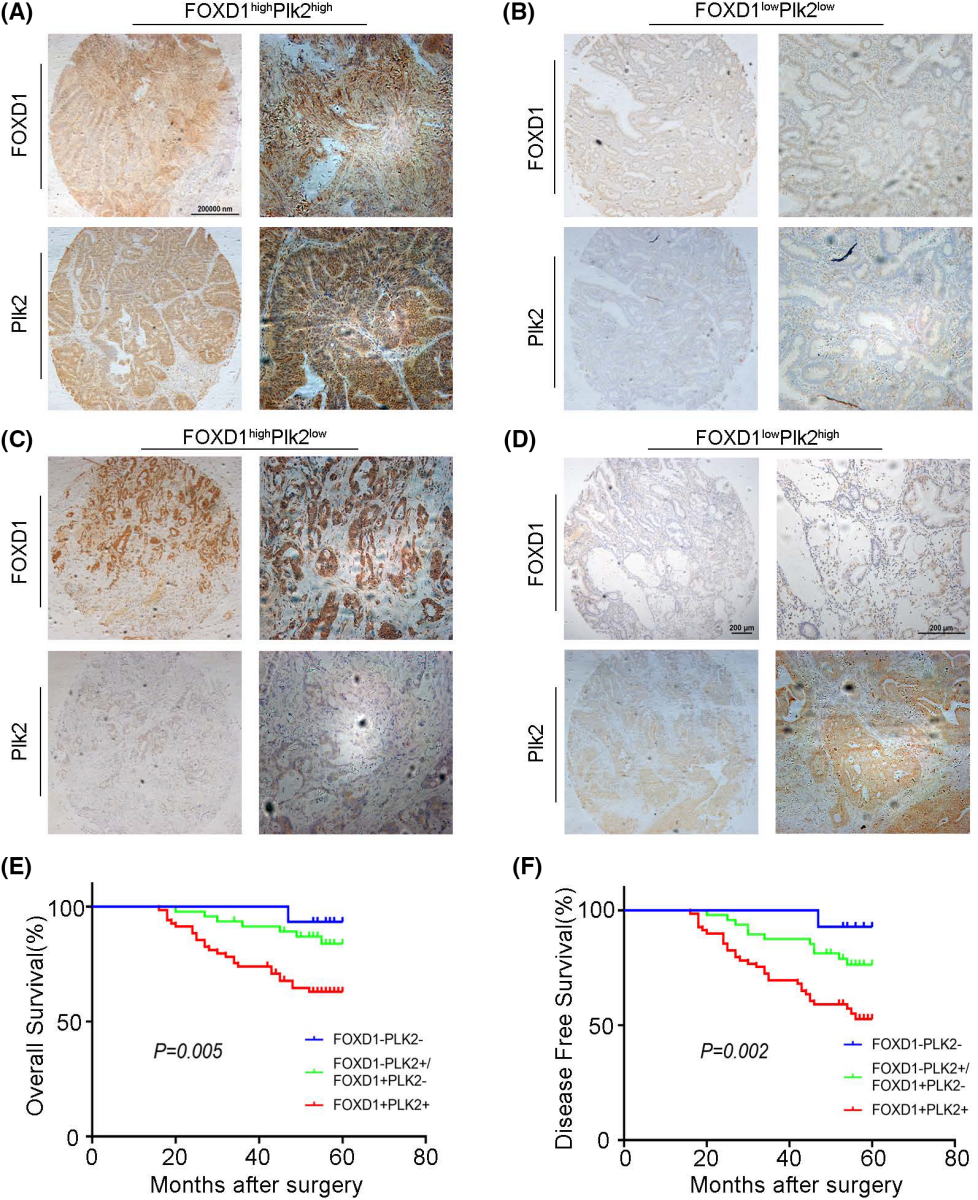
Combination of FOXD1 and Plk2 to predict prognosis of CRC patients. A to D: expression of FOXD1 and Plk2 in CRC tissues, A‐FOXD1^high^/Plk2^high^; B‐FOXD1^high^/Plk2^low^; C‐FOXD1^low^/Plk2^high^; and D‐FOXD1^low^/Plk2^low^. E&F: Kaplan–Meier analysis of OS(E) and DFS(F) in 4 groups

Plk2 is a member of the Polo‐like kinases (Plk) family. Previously, we have found that Plk2 had a high expression in tumour tissues and might be an independent prognostic marker for CRC patients.^9^ Therefore, we analysed the relationship between the expression of FOXD1 and Plk2 in our cohort of 131 CRC cases. Pearson's correlation analysis indicated a strong correlation between the expression of FOXD1 and Plk2 (*r* = 4.807, *p* = 0.028). Next, we analysed these two proteins as a whole and categorized the overall cohort into three groups: positive (FOXD1^high^/Plk2^high^), intermediate (FOXD1^high^/Plk2^low^, FOXD1^low^/Plk2^high^) and negative staining groups (FOXD1^low^/Plk2^low^) (Figure 6A–D). Kaplan–Meier survival analysis revealed that positive staining group showed poorer prognosis in contrast to intermediate and negative staining groups (Figure 6E,F), and the two‐factor prognostic score (FOXD1/Plk2) overweighed single FOXD1 factor in the prediction of both OS and DFS (*p* = 0.005 and *p* = 0.002). In addition, we divided FOXD1high/Plk2low and FOXD1low/Plk2high into two independent groups and pictured Kaplan–Meier survival curve (Supporting Information Figure S1). In this figure, thereis no statistically significant difference between FOXD1high/Plk2low and FOXD1low/Plk2high.

On this basis, we further created a unique predictive model for postoperative CRC patients in the merge of the TNM staging system and the two‐factor prognostic score (FOXD1/PLK2) (Table 5). The advanced model involving the TNM stage and FOXD1/PLK2 expression showed a higher C‐index (OS:0.772 vs 0.744 vs 0.686, DFS:0.762 vs 0.738 vs 0.687) and lower AIC (OS: 280 vs 287 vs294, DFS:360 vs 369 vs 378) than the one based on TNM stage and FOXD1 expression and that of the TNM stage alone in both OS and RFS. Taken together, the combination of FOXD1 and Plk2 allows us to predict the survival of post‐surgical CRC patients more accurately, adding to the prognostic model based only on the TNM stage.

**TABLE 5:** Comparison of the prognostic accuracy of TNM staging and the combined model

Model	OS			DFS		
	C‐index	95%CI	AIC	C‐index	95%CI	AIC
TNM	0.686	0.669–0.704	294.3	0.687	0.671–0.703	377.66
FOXD1	0.597	0.583–0.611	306.91	0.583	0.569–0.60	396.56
TNM + FOXD1	0.744	0.725–0.763	286.67	0.738	0.72–0.756	368.99
TNM + FOXD1/PLK2	0.772	0.753–0.791	280.12	0.762	0.744–0.78	359.80

Abbreviations: AIC, Akaike information criterion; C‐index, concordance index; foxD1, FOXD1 expression; FOXD1/TLK2, a combination of FOXD1 and TLK2 expression TNM, 8th edition AJCC TNM staging system.

### 3.7 Prognostic nomogram model of FOXD1 with or without Plk2 in CRC patients

**FIGURE 7:**
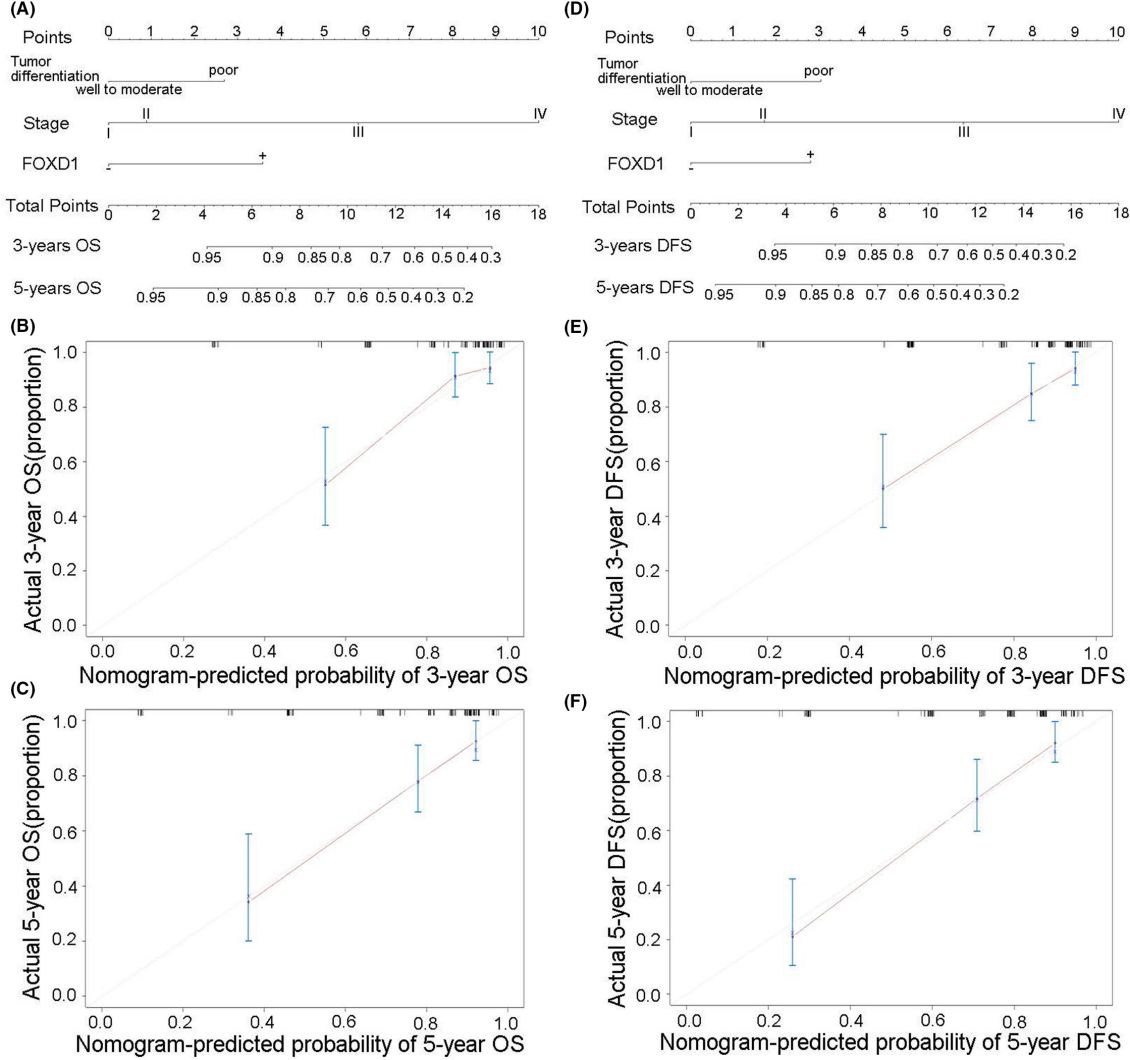
The prediction of prognosis in CRC patients using the nomogram model. A, B&C: OS prediction and calibration plots and D–F: DFS prediction and calibration plots

**FIGURE 8:**
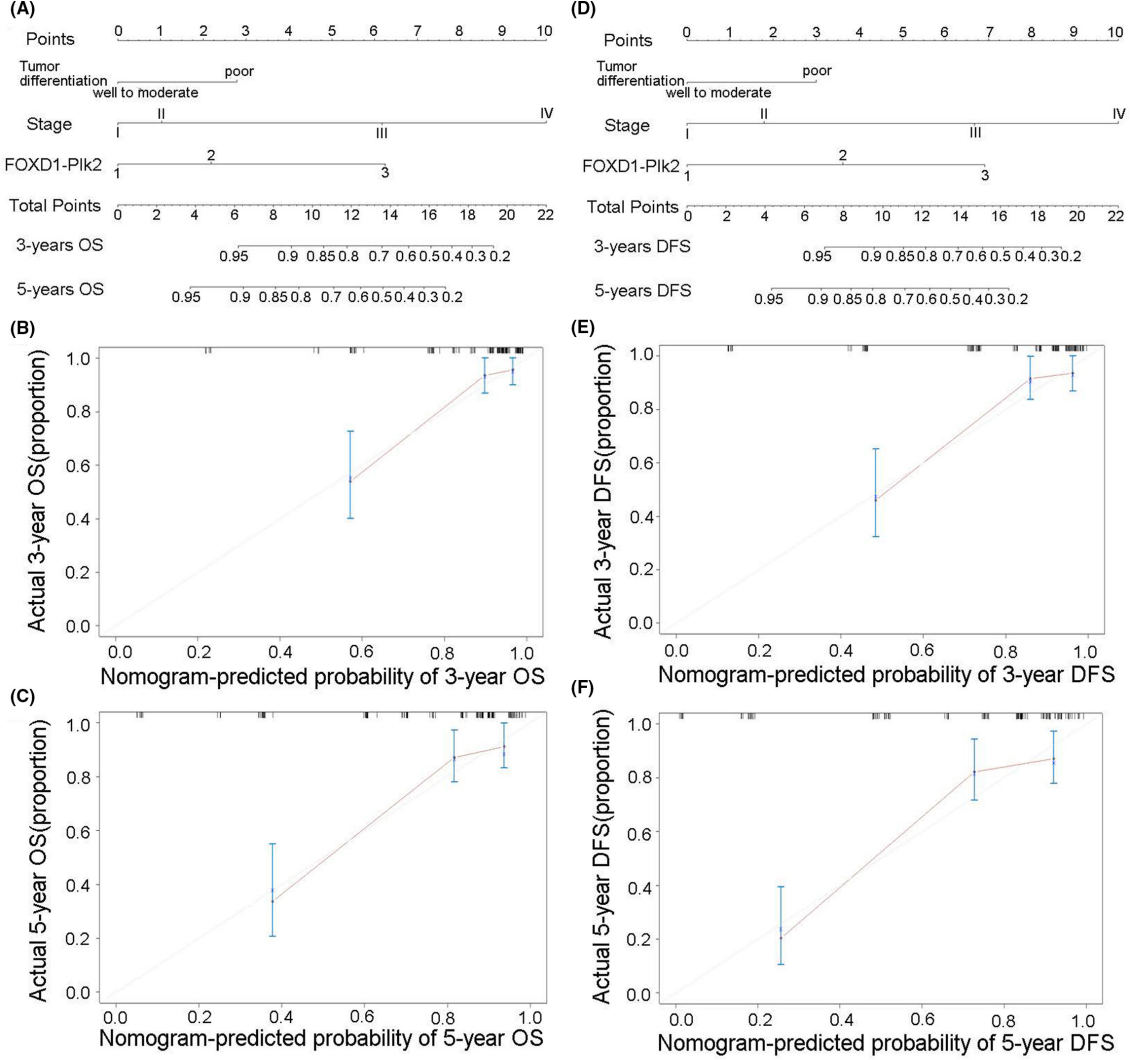
Nomogram model for the prediction of prognosis with a combination of FOXD1 and Plk2 in CRC patients. A–C combination prediction and calibration plots for OS; D–F: combination prediction and calibration plots for DFS

FOXD1 expression, TNM stage and tumour differentiation were proven to be independent factors for both OS and DFS through the previous analysis. On this basis, nomogram model was constructed in prediction of OS and DFS at 3 and 5 years after surgery in CRC patients (Figure 7 A,D). An optimal consistency was manifested in the calibration plot which was used to verify the difference between the theoretical and actual prediction probability of OS and DFS at 3 or 5 years after surgery (Figure 7B,C,E,F, respectively). We also constructed a nomogram model with a combination of FOXD1 and Plk2 to predict OS and DFS at 3 and 5 years (Figure 8A,D). Similarly, the calibration plot also manifested an optimal consistency (Figure 8B,C,E,F, respectively). In addition, C‐index analysis demonstrates that the combination of TNM and FOXD1 has more accuracy than the TNM stage or FOXD1, respectively (C‐index = 0.744 vs C‐index = 0.686 or 0.597). Moreover, a combination of TNM, FOXD1 and Plk2 has the most powerful prediction ability (C‐index = 0.772).

## 4 DISCUSSION

CRC is the third leading cause of death in terms of malignancy. Therefore, novel diagnostic prediction markers are necessary to predict survival and decrease the mortality of CRC patients. Transcription factors are engaged in a huge number of biological processes associated with cancer such as DNA replication, DNA repair, chromosome condensation or DNA unwinding and of course DNA et al. Additionally, transcription factors account for about 20% of all oncogenes identified so far.^11^ Here in our study, we identified a total of 3559 DEGs (3372 upregulated genes and 187 downregulated genes). KEGG enrichment analysis demonstrated that the cell cycle is the most significantly enriched pathway in CRC. Among these DEGs, 66 upstream transcription factors (62 upregulated and 4 downregulated TFs) are included. E2F6, PLAG1, EGR3, FOXD1, KLF4, NR2F1, TFAP2A, NFYB, TFAP2C and TFDP1 are the top 10 TFs covering the most downstream DEGs. FOXD1 was the most DEGs in the 10 TFs.

LASSO‐Cox regression analysis reveals that FOXD1, TFAP2A, TFAP2C and KLF4 are the most statistically significant prognostic factors. In addition, Kaplan–Meier survival curve and multivariate COX regression analysis of these four TFs indicated that FOXD1 is an independent risk prognostic factor and KLF4 is an independent protective prognostic factor associated with OS. Therefore, we choose FOXD1 as a target TF for further analysis and verification.

The function of FOXD1 involved in cancer evolution has been revealed in nasopharyngeal carcinoma, non‐small cell lung cancer and breast cancer.^6^, ^7^, ^8^ However, FOXD1 acts as a predictor of survival in CRC has not been fully elucidated. As is shown above, FOXD1 is verified to be high‐expressed in CRC tissues in contrast to peritumoral normal tissues, which is in concordance with the report on other cancer types.^7^, ^8^ FOXD1 is mainly expressed in the cytoplasm of cancer cells. We also found that high expression of FOXD1 was linked with tumour differentiation. Fengping et al. report that high expression of FOXD1 is associated with tumour size except for tumour differentiation.^12^ But in our study, no correlation between FOXD1 expression and tumour size was found. This means that a cohort with more patients is necessary to solve the issue.

Univariate Cox regression analysis demonstrates that TNM stage, tumour differentiation and positive FOXD1 expression are prognostic risk factors for OS and DFS. Moreover, multivariate COX regression analysis further demonstrated that TNM stage, tumour differentiation and positive FOXD1 expression are respectively independent risk factors in CRC patients. Additionally, patients with high expression of FOXD1 show poor prognosis both in OS and DFS. Moreover, TNM stage, tumour differentiation and positive FOXD1 expression can be used to predict 3 and 5 years of survival in the Nomogram model. These findings suggest that FOXD1 is an unfavourable prognostic factor for disease progression and patients’ prognosis.

Plk2 belongs to the Plk family which is a highly conserved family of serine‐threonine kinases engaged in the regulation of the cell cycle and cellular response to stresses such as DNA damage.^13^ In addition, we have proven that high expression of Plk2 can promote CRC progression in our previous study.^9^ Therefore, we combined FOXD1 and Plk2 to analyse their relationship with patients’ prognosis. Results suggested that FOXD1^high^/Plk2^high^ group has worse OS and DFS in contrast to FOXD1^high^/Plk2^low^ or FOXD1^low^/Plk2^high^, and FOXD1^low^/Plk2^low^ groups. This implies that a combination of FOXD1 and Plk2 may have a more powerful predictive ability for unfavourable prognosis. Moreover, TNM stage, tumour differentiation and positive FOXD1 expression are capable of predicting 3 and 5 years of survival in the Nomogram model.

Taken together, we identified FOXD1 as one of the transcription factors covering most downstream DEGs which indicates FOXD1 may play an important role in CRC. Next, we verified that FOXD1 expression is associated with CRC patients’ pathological differentiation and invasion. Additionally, FOXD1 is an unfavourably predictor of CRC patients’ prognosis. Moreover, FOXD1 and Plk2 help stratify patients at high risk. Therefore, FOXD1 can be used as a new molecule to predict prognosis in CRC patients. A combination of FOXD1 and Plk2 has a more powerful prediction ability for survival. The major limitations of our study are the relatively small number of CRC patients enrolled. A multi‐center, prospective study is in demand to guarantee these results in a larger population in the future.

## AUTHOR CONTRIBUTION

**Yaping Zong:** Conceptualization (equal); Data curation (lead); Investigation (lead). **Yiming Miao:** Methodology (lead). **Wenchang Li:** Project administration (equal); Software (equal). **Minhua Zheng:** Supervision (equal). **Zhuoqing Xu:** Formal analysis (equal). **Han Gao:** Project administration (lead). **Wenqing Feng:** Visualization (lead). **Zifeng Xu:** Writing – original draft (equal). **Jingkun Zhao:** Supervision (equal); Writing – original draft (equal); Writing – review & editing (lead). **Lifei Shen:** Supervision (equal). **Aiguo Lu:** Conceptualization (lead); Supervision (lead); Writing – original draft (lead); Writing – review & editing (lead).

## CONFLICT OF INTEREST

The authors display no conflict of interest.

## Supporting information

Fig S1‐S2

Table S1‐S3
