# Supplementary figures and images for: Combination of FOXD1 and Plk2: A novel biomarker for predicting unfavourable prognosis of colorectal cancer
Source: J Cell Mol Med. 2022 May 17;26(12):3471–82. doi: 10.1111/jcmm.17361 (PMC9189346; doi:10.1111/jcmm.17361)

Supplementary Figure 1

OS

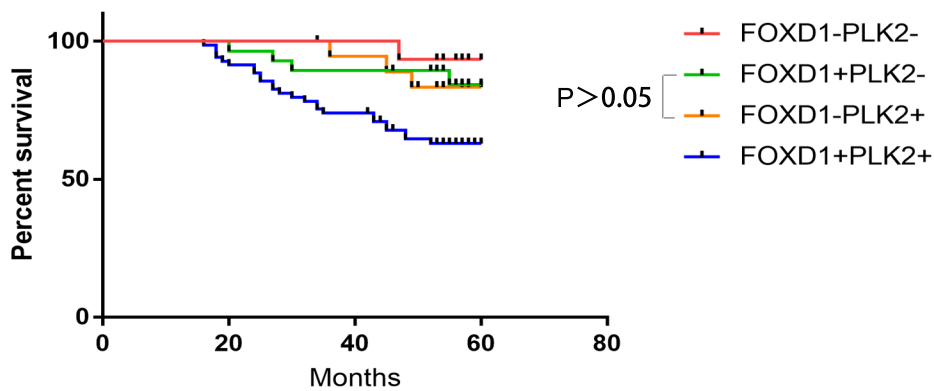

DFS

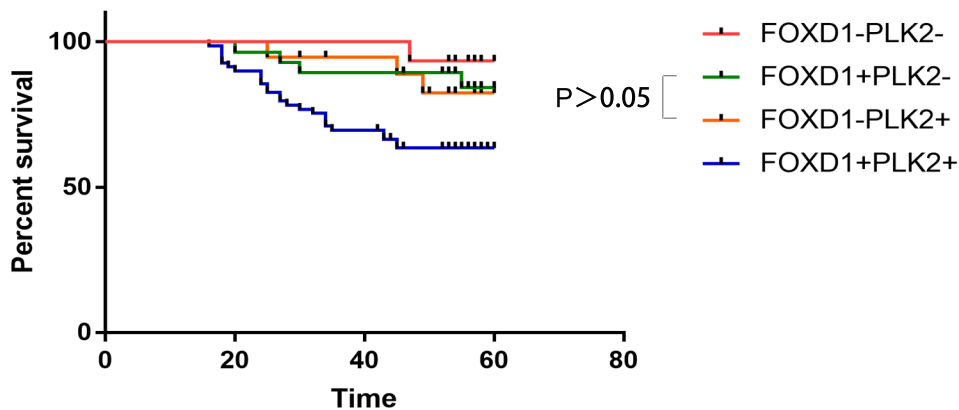

A

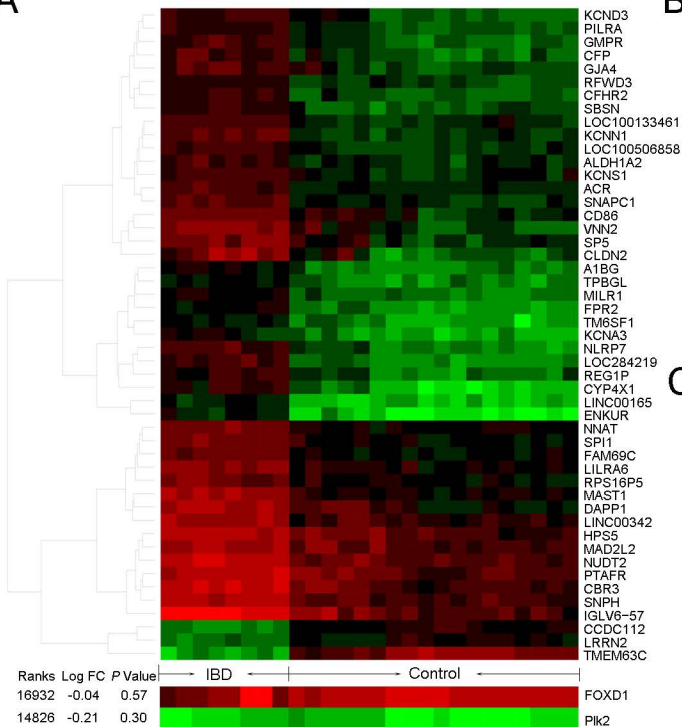

B

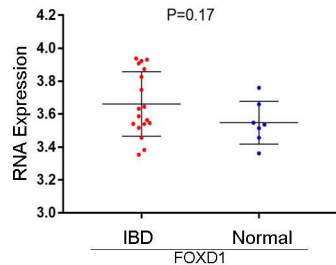

C

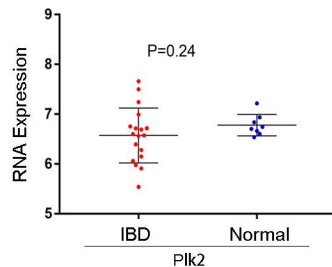

Supplement: Supplementary file 1 — Fig S1‐S2 [file JCMM-26-3471-s001.pdf]
