# Supplementary material for: Combination of FOXD1 and Plk2: A novel biomarker for predicting unfavourable prognosis of colorectal cancer
Source: J Cell Mol Med. 2022 May 17;26(12):3471–82. doi: 10.1111/jcmm.17361 (PMC9189346; doi:10.1111/jcmm.17361)
Supplement: Supplementary file 2 — Table S1‐S3 [file JCMM-26-3471-s002.docx]

**Table S1 Basic clinical characteristics of Seven GEO datasets used in our study**

| GEO Datasets | Platforms | Tumor vs. Normal | Time | Authors | Species |
| --- | --- | --- | --- | --- | --- |
| GSE23878 | GPL570 | 35 vs. 24 | 2011 | Uddin S, et al. | *Homo sapiens* |
| GSE4107 | GPL570 | 12 vs. 10 | 2007 | Hong Y, et al. | *Homo sapiens* |
| GSE41328 | GPL570 | 10 vs. 10 | 2006 | Lin G, et al. | *Homo sapiens* |
| GSE33113 | GPL570 | 90 vs. 6 | 2011 | Medema, et al. | *Homo sapiens* |
| GSE18088 | GPL570 | 53 vs. 0 | 2011 | Gröne， et al. | *Homo sapiens* |
| GSE30540 | GPL570 | 35 vs. 0 | 2012 | Watanabe, et al. | *Homo sapiens* |
| GSE31595 | GPL570 | 37 vs. 0 | 2011 | Thorsteinsson, et al. | *Homo sapiens* |

**Table S2 Top 10 most significantly enriched KEGG pathways**

| KEGG ID | Description | FDR | Genes |
| --- | --- | --- | --- |
| hsa04110 | Cell cycle | 3.62E-13 | ANAPC10/ANAPC11/ATR/BUB1/BUB1B/BUB3/CCNA2/CCNB1/CCND1/CCND2/CCNE1/CCNE2/CDC16/CDC20/CDC23/CDC25A/CDC25B/CDC25C/CDC27/CDC45/CDC6/CDC7/CDK1/CDK2/CDK4/CDK6/CDK7/CDKN1B/CHEK1/CHEK2/DBF4/E2F1/E2F3/E2F5/ESPL1/HDAC2/MAD1L1/MAD2L1/MAD2L2/MCM2/MCM3/MCM4/MCM5/MCM6/MCM7/MYC/ORC1/ORC5/ORC6/PCNA/PKMYT1/PLK1/PTTG1/RB1/RBL1/SKP2/SMC3/TFDP1/TTK/WEE1/YWHAB/YWHAG |
| hsa03030 | DNA replication | 4.29E-10 | FEN1/LIG1/MCM2/MCM3/MCM4/MCM5/MCM6/MCM7/PCNA/POLA1/POLD1/POLD2/POLE2/POLE3/PRIM1/PRIM2/RFC2/RFC3/RFC4/RFC5/RNASEH1/RNASEH2A/RNASEH2B/RPA2/RPA3/SSBP1 |
| hsa03013 | RNA transport | 1.23E-07 | ALYREF/DDX20/EIF1AX/EIF2B3/EIF2S2/EIF2S3/EIF3B/EIF3C/EIF3E/EIF3G/EIF3H/EIF3I/EIF3J/EIF4A3/EIF4EBP1/EIF4G1/EIF5B/FUS/GEMIN2/GEMIN4/GEMIN5/GEMIN8/KPNB1/MAGOHB/NCBP2/NDC1/NMD3/NUP107/NUP133/NUP155/NUP160/NUP205/NUP210/NUP35/NUP37/NUP43/NUP50/NUP62/NUP85/NUP98/NUPL1/NXT1/NXT2/PABPC1L/PABPC3/PAIP1/POM121/POM121C/POP1/POP7/PRMT5/RAE1/RAN/RANGAP1/RNPS1/RPP40/SEC13/SEH1L/STRAP/TACC3/TGS1/THOC2/THOC6/TRN |
| hsa03430 | Mismatch repair | 0.000630629 | EXO1/LIG1/MSH2/MSH6/PCNA/POLD1/POLD2/RFC2/RFC3/RFC4/RFC5/RPA2/RPA3/SSBP1 |
| hsa03050 | Proteasome | 0.001250621 | ADRM1/IFNG/POMP/PSMA2/PSMA5/PSMA7/PSMB2/PSMB3/PSMB9/PSMC3/PSMC4/PSMC6/PSMD12/PSMD13/PSMD14/PSMD3/PSMD7/PSME3/PSME4/PSMF1/SHFM1 |
| hsa03040 | Spliceosome | 0.001250621 | ALYREF/CDC40/CHERP/CTNNBL1/EIF4A3/FUS/HNRNPA1/HNRNPU/HSPA6/LSM2/LSM5/LSM6/LSM7/MAGOHB/NCBP2/PPIH/PPIL1/PRPF19/PRPF31/PRPF4/PRPF40A/PRPF6/PUF60/RBMXL1/RP9/SF3A2/SF3B3/SMNDC1/SNRNP200/SNRNP40/SNRNP70/SNRPA/SNRPB/SNRPB2/SNRPC/SNRPD1/SNRPD2/SNRPD3/SNRPE/SNRPF/SNRPG/SRSF10/SRSF6/THOC2/TRA2B/U2AF2/U2SURP/WBP11/XAB2 |
| hsa03420 | Nucleotide excision repair | 0.001447819 | CDK7/CETN2/CUL4A/CUL4B/ERCC2/GTF2H3/GTF2H4/LIG1/MNAT1/PCNA/POLD1/POLD2/POLE2/POLE3/RAD23A/RFC2/RFC3/RFC4/RFC5/RPA2/RPA3 |
| hsa03440 | Homologous recombination | 0.001611782 | BABAM1/BARD1/BLM/BRCA1/BRCA2/BRIP1/NBN/PALB2/POLD1/POLD2/RAD51D/RAD54B/RAD54L/RBBP8/RPA2/RPA3/SHFM1/SSBP1/TOPBP1 |
| hsa03008 | Ribosome biogenesis in eukaryotes | 0.001843687 | BMS1/CIRH1A/CSNK2A1/CSNK2A2/EIF6/EMG1/FBL/GAR1/GNL3L/GTPBP4/HEATR1/NAT10/NHP2/NMD3/NOB1/NOP58/NXT1/NXT2/POP1/POP7/RAN/RBM28/RCL1/RIOK1/RPP40/RRP7A/SPATA5/TBL3/TCOF1/UTP14A/UTP15/UTP18/WDR3/WDR36/WDR43/WDR75/XPO1/XRN2 |
| hsa03410 | Base excision repair | 0.002663068 | APEX1/APEX2/FEN1/HMGB1/LIG1/NEIL3/NTHL1/PARP1/PARP2/PARP3/PCNA/POLD1/POLD2/POLE2/POLE3/UNG |

**Table S3. Comparison of the expression of Plk2 in colon and rectum cancer**

| Clinicopathologic Parameters | | Plk2 expression | | | *P* Value |
| --- | --- | --- | --- | --- | --- |
|  |  | Case (n=131) | Positive | Negative |  |
| Location | Rectum | 49 | 31 | 18 | 0.556 |
|  | Colon | 82 | 56 | 26 |  |
